# Coexpression of Helios in Foxp3^+^ Regulatory T Cells and Its Role in Human Disease

**DOI:** 10.1155/2021/5574472

**Published:** 2021-06-22

**Authors:** Wen-qing Yu, Ning-fei Ji, Cheng-jing Gu, Yan-li Wang, Mao Huang, Ming-shun Zhang

**Affiliations:** ^1^Department of Infectious Diseases, Taizhou People's Hospital Affiliated to Nantong University, Taizhou, Jiangsu 225300, China; ^2^Department of Respiratory and Critical Care Medicine, The First Affiliated Hospital of Nanjing Medical University, Nanjing, Jiangsu 210029, China; ^3^Department of Pharmacy, Taizhou People's Hospital Affiliated to Nantong University, Taizhou, Jiangsu 225300, China; ^4^Department of Immunology, Nanjing Medical University, Nanjing, Jiangsu 211166, China; ^5^NHC Key Laboratory of Antibody Technique, Nanjing Medical University, Nanjing, Jiangsu 211166, China

## Abstract

Regulatory T cells (Tregs) expressing the Foxp3 transcription factor are indispensable for the maintenance of immune system homeostasis. Tregs may lose Foxp3 expression or be reprogrammed into cells that produce proinflammatory cytokines, for example, Th1-like Tregs, Th2-like Tregs, Th17-like Tregs, and Tfh-like Tregs. Accordingly, selective therapeutic molecules that manipulate Treg lineage stability and/or functional activity might have the potential to improve aberrant immune responses in human disorders. In particular, the transcription factor Helios has emerged as an important marker and modulator of Tregs. Therefore, the current review focuses on recent findings on the expression, function, and mechanisms of Helios, as well as the patterns of Foxp3^+^ Tregs coexpressing Helios in various human disorders, in order to explore the potential of Helios for the improvement of many immune-related diseases. The studies were selected from PubMed using the library of the Nanjing Medical University in this review. The findings of the included studies indicate that Helios expression stabilizes the phenotype and function of Foxp3^+^ Tregs in certain inflammatory environments. Further, Tregs coexpressing Helios and Foxp3 were identified as a specific phenotype of stronger suppressor immune cells in both humans and animal models. Importantly, there is ample evidence that Helios-expressing Foxp3^+^ Tregs are relevant to various human disorders, including connective tissue diseases, infectious diseases, solid organ transplantation-related immunity, and cancer. Thus, Helios^+^Foxp3^+^CD4^+^ Tregs could be a valuable target in human diseases, and their potential should be explored further in the clinical setting.

## 1. Introduction

Regulatory T cells or Tregs, which form a distinct subset of CD4^+^ T cells, were once termed suppressor T cells, as they were found to restrict antigen-specific T cell responses (based on the results of Gershon and Kondo's experiments in the 1960s and 1970s) [1, 2]. Tregs play an essential role in the negative regulation of excessive inflammatory responses, the establishment and maintenance of self-tolerance, and the impaired anticancer effects of conventional and novel immunotherapy agents [3–5]. Their generation, induction, phenotype maintenance, and functional activity, especially in local microenvironments, have always been a hot research topic. In 2003, Foxp3 was identified as the most definitive marker associated with Treg differentiation and function, and it facilitated the identification of a specialized subpopulation of CD4 T cells [6–9]. In addition to the classic CD4^+^ Tregs, a diverse regulatory subpopulation of lymphocytes has been identified, including IL-10-Tr1, TGF-*β*-Th3, CD8^+^ Tregs, TCR*αβ*^+^CD4^−^CD8^−^ Tregs, IL-10-Bregs, *γδ*-Tregs, and regulatory innate lymphoid cell (ILCregs) [10–13]. The majority of Tregs are characterized by high expression of CD25 (IL-2R-*α* chain), presence of Foxp3, and low or no expression of CD127 (IL-7R-*α* chain) [14]. However, markers or combinations of markers that are specific to certain *bona fide* Tregs with greater immunosuppressive capacities still need to be identified [15].

With the advances in research in recent years, an increasing number of factors have been found to be involved in Treg-mediated suppression, including cytokines, surface molecules, transcription factors, metabolic pathways, and genetic modifications [16, 17]. Among these factors, Helios has emerged as an important marker and functional modulator of Tregs, and it may even have potential as a molecular target for improving immune-related diseases [18, 19]. In this review, we gather recent results relating to the functions, regulatory pathways, and molecular mechanisms of Helios, as well as the significant changes in Foxp3^+^ Tregs coexpressing Helios in human disorders, in an attempt to gain a better understanding of the immune microenvironment in Treg-mediated diseases and the potential therapeutic benefits of Helios.

## 2. Functional Characterization of Helios

Helios (IKZF2), a member of the *Ikaros* transcription factor family (which also comprises Ikaros (IKZF1), Aiolos (IKZF3), Eos (IKZF4), and Pegasus (IKZF5)), was originally identified as a novel dimerization partner of Ikaros [20, 21]. Helios and its protein isoforms are encoded by transcript variants of the Helios gene (*IKZF2*) located on human chromosome 2q34. Similar to other members of the Ikaros family, Helios possesses four highly conserved N-terminal and two C-terminal zinc finger motifs, which interact with gene regulatory elements through their DNA-binding regions and form various homodimers, heterodimers, and multimers that repress or activate the transcription of specific genes, especially those involved in the differentiation and development of lymphocytes and other hematopoietic cells [22–24]. The expression of Helios is specific to multipotent lymphoid progenitors and T-lineage cells at different developmental stages; in contrast, Helios expression is absent in most B-lineage cells [25, 26]. The molecular mechanisms underlying the cell-specific distribution pattern of Helios remain to be elucidated.

The reviewed studies indicate that Helios silencing may be critical for the normal development and function of B cells [26]. Although the effects are small, ectopic levels of Helios expression clearly promote B cell abnormalities [25, 27]. One study reported that transgenic expression of full-length Helios in B cells resulted in the development of metastatic B cell lymphoma in mice [25]. The B cells from Helios transgenic animals were highly reactive to antigenic stimulation, which exhibited the increased cell receptor-mediated proliferation and the improved survival in the bone marrow and spleen [25]. In contrast to its expression in B cells and some antigen-matured T cells, Helios is preferentially and highly expressed by Foxp3^+^ T cells and is required for stable inhibitory activity of Tregs. In fact, Helios has been shown to be a marker of stable phenotype and function in Tregs and is also closely associated with demethylation of a Treg-specific demethylated region (TSDR) of the Foxp3 locus [28, 29].

In human and mouse models, it has been recently observed that Helios^+^ Tregs have greater immunosuppressive activity than Helios^−^ Tregs [19, 30, 31]. Importantly, Helios knockdown in human Tregs was found to decrease Foxp3 expression and impair its immunosuppressive functions [31]. Interestingly, in contrast to Foxp3^+^Helios^+^ T cell, the Foxp3^+^Helios^−^ T cell has the ability to secrete proinflammatory cytokines such as IL-2, IL-17, and IFN-*γ* [32, 33]. Nevertheless, under inflammatory conditions, Helios is important for the phenotypic and functional stability of Foxp3^+^CD4^+^ and CD8^+^ Tregs [28, 31]. With regard to its mechanism, Helios not only directly binds to the promoter of *Foxp3* and augments *Foxp3* transactivation but also silences the IL-2 gene promoter to contribute to the development and stability of Tregs [31, 34].

In the tumor microenvironment, selective deletion of Helios contributes to the unstable phenotype of Tregs and their conversion into T effector cells, and this improves antitumor immunity in tumor-bearing animals [35]. Additionally, ectopic Helios expression also affects the interaction between Tregs and tumor cells. In childhood B cell precursor acute lymphoblastic leukemia, increased Helios expression in Tregs facilitates the infiltration and metastasis of leukemia stem cells by elevating Vascular endothelial growth factor A (VEGFA) expression and Vascular endothelial growth factor receptor 2 (VEGFR2) activity and modulates leukemia cell apoptosis by promoting the expression of the antiapoptosis protein Bcl-2 [36]. Therefore, Helios might have the potential to modulate Treg-dependent resistance to antitumor responses. However, as Helios is an intracellular transcription factor, functional studies on it are limited by the difficulty involved in isolating viable cell subsets.

## 3. Induction and Regulation of Helios Expression

Under *in vitro* conditions, Helios expression can be induced or inhibited by cytokines and several signaling pathways in human and murine CD4^+^ T cells. Both TGF-*β* signaling and intrinsic Foxp3 expression are required for Helios expression in murine induced Tregs [37, 38]. Helios functions in synergy with Foxp3 to enhance the expression of Treg-related molecules and the suppressive function of induced Tregs [37]. However, retrovirus-mediated induction of Foxp3 in murine CD4^+^ T cells did not significantly increase Helios expression in the absence of TGF-*β* [37]. This probably means that the expression of Helios does not absolutely depend on Foxp3.

In addition to Foxp3, IL-2 was also found to promote the expression of Helios via induction of STAT5 phosphorylation, as well as control the expression of IL-2R, thereby promoting IL-2-driven signaling and contributing to the maintenance of Helios expression [28, 39–41]. More recent studies have shown that Tregs in aged mice with declining levels of IL-2 also showed high expression of Helios [41, 42]. Thus, IL-2 and/or other factors may also change Helios expression in Tregs.

In contrast to the findings for IL-2, IL-6 was found to suppress TGF-*β*-induced Helios expression through STAT3-dependent signaling [37]. Importantly, the expression of Helios in CD4^+^ T cells was significantly increased in rheumatoid arthritis (RA) patients who were successfully treated with tocilizumab (TCZ), a humanized anti-IL-6R antibody [37]. It has also been shown that the proliferation of Helios^+^ and Helios^−^ Tregs from healthy individuals in *ex vivo* cultures could be regulated by CD16^+^ and CD14^+^CD16^−^ monocyte subsets via IL-12 and TNF-*α*, respectively [43]. Thus, Helios expression during Treg development might be controlled by diverse, but specific, inflammatory cytokines in animals and patients with immune disorders.

Some other molecules have also been proposed to be involved in the modulation of Helios expression. For example, stimulation with a TNFR-2 agonist monoclonal antibody (TNFR-2 agonist mAb), along with rapamycin, was found to enhance the expression of Helios and Foxp3 in human Tregs under *ex vivo* conditions, as well as promote Treg expansion [44]. Additionally, 1,25-dihydroxyvitamin D3 alone, or in synergy with estrogen, induced Helios expression and increased the number of CD4^+^Helios^+^Foxp3^+^ Tregs in a Vdr-dependent manner [45, 46]. Further, a previous study demonstrated that the antitumor effect of GITR was associated with its *in vivo* effects on Foxp3^+^CD4^+^ Tregs via its antibody DTA-1, which acts as a GITR agonist [47]. Ligation of GITR caused a significant reduction in the number of intratumor Tregs and loss of Foxp3 expression [47]. Importantly, DTA-1 treatment also resulted in reduced Helios expression, which was accompanied by upregulation of the effector T cell transcription factors ROR-*γ*t, T-bet, and Eomes [47]. In another paper by Kim and colleagues, too, it was confirmed that the agonistic anti-GITR antibody, which triggers downregulation of Helios, promotes the conversion of intratumoral Tregs into effector T cells [35, 41]. Additionally, soluble GITR present in the peripheral blood of patients with myasthenia gravis also promoted Helios expression and enhanced Treg function independently of membrane GITRL [48]. Thus, those studies indicate that Helios expression is also influenced by surface receptors and other molecules.

Loss of Foxp3 expression frequently occurs in *in vitro* expanded Tregs. However, addition of phosphorothioated random oligonucleotides during expansion culture results in the stabilization of Foxp3 expression and prolonged stabilization of the Foxp3(+)Helios(+) subpopulation [45, 46]. With regard to the underlying mechanism, it has been shown that these oligonucleotides stabilize Foxp3 and Helios expression with the help of sterile *α* motif histidine-aspartate-domain containing protein 1 in human Tregs at the posttranscriptional level [49]. Another related and interesting finding was reported in a recent study: Treg phenotype and function during *in vitro* expansion are also affected by culture temperature. That is, Tregs cultured under a mildly hypothermic temperature of 33°C exhibit robust proliferation; high and stable expression of Foxp3, CD25, and Helios; and remarkable demethylation of TSDR [50]. Thus, in addition to Foxp3 and Helios, temperature also has an impact on Treg stability.

## 4. Helios-Expressing Foxp3^+^ Tregs and Human Disease

### 4.1. Autoimmune Diseases

Reports on the quantitative and qualitative features of Tregs in patients with various autoimmune diseases, such as systemic lupus erythematosus (SLE), are controversial. The combination of Helios and Foxp3 is known as a valid marker of functional Tregs. In the context of SLE, Foxp3^+^Helios^+^ Tregs from peripheral blood mononuclear cells (either freshly extracted or extracted after 4 h of stimulation for cytokine production) were not reduced in patients with active SLE [51]. Furthermore, in patients with moderately-to-highly active SLE, these cells were maintained at normal levels after correction for the total CD4 cell count. In addition, it was also shown that the Foxp3^+^Helios^−^ subset may contain cytokine-producing conventional T cells, which produce IL-2 or IFN-*γ*, but the Foxp3^+^Helios^+^ population comprised only non-cytokine-producing cells in both SLE patients and healthy controls [51]. In another study, too, it was shown that Foxp3^+^Helios^+^ Tregs, unlike Foxp3^+^Helios^−^ cells, were found in high numbers in SLE patients and were positively correlated with disease activity, but this was not observed in systemic sclerosis (SSc) or RA [51, 52]. This might be associated with the presence of self-antigens in active SLE, including DNA nucleotides, as oligodeoxynucleotides were found to stabilize *in vitro* Helios expression in Tregs [32]. This explains Helios-induced stabilization of Tregs in SLE, but the corresponding mechanism in SSc or RA is unclear. Although, similar to the observation in healthy controls, Helios-expressing Foxp3^+^ Tregs in SLE patients did not produce effector cytokines and possessed a highly demethylated TSDR, they still failed to fully protect the host from intense self-reactive B and T cell responses. This could mean that the activity of Foxp3^+^Helios^+^ Tregs in different microenvironments remains to be identified.

RA is a chronic inflammatory autoimmune disease that is characterized by immune dysregulation. Evidence shows that Tregs are involved in the inflammatory process in RA. A recent study reported that Foxp3 gene expression and TSDR demethylation were significantly lower in newly diagnosed RA patients than in healthy subjects, but Helios gene expression in peripheral blood was higher in the RA patients [53]. It is speculated that both epigenetic modifications and Helios expression may be involved in the development of RA via their effects on Treg stability. Additionally, tissue-specific Tregs differ from Tregs that are in circulation: Helios expression in Foxp3^+^CD4^+^ T cells was more abundant in synovial fluid from rheumatic joints than in peripheral blood [54]. Under *in vitro* conditions, joint-derived Foxp3^+^Helios^+^ Tregs lacked IFN-*γ*, TNF, and IL-10 production, while the Fxop3^+^Helios^−^ subset did not. In addition, Foxp3^+^Helios^+^ Tregs exhibited the features of classical Tregs, including expression of CD25 and cytotoxic T lymphocyte-associated antigen (CTLA-4) and demethylation of TSDR. Thus, the aberrant expression of Helios in Foxp3^+^CD4^+^ T cells may be associated with the pathogenesis of RA.

### 4.2. Allogeneic Organ Transplant

Diverse Treg subsets have been observed in patients who exhibit allogeneic graft tolerance, and these cells contribute to the establishment and maintenance of long-term allograft tolerance by inhibiting the alloreactivity of T and B lymphocytes as well as natural killer cells [55–57]. In liver transplantation patients, Helios and Foxp3 expression, the ratio of LAP^+^ Tregs to CD45RA^−^HLA^−^DR^+^ Tregs, and the degree of Foxp3 TSDR demethylation were higher in the tolerant group than in the nontolerant group [57]. It is worth noting that partial or unstable demethylation of TSDR in the Foxp3 gene of Tregs is often predictive of poor or gradual loss of Treg suppressive function [58]. Moreover, in tolerant patients who underwent liver transplantation, several miRNAs, including miR-155, miR-146a, and miR-24, were more highly expressed and associated with Treg-mediated tolerance than in nontolerant patients [57]. Interestingly, the proportion of Helios^+^ Tregs in the peripheral blood of tolerant patients with combined liver-kidney transplantation was higher than that in patients who underwent liver or kidney transplant alone [59]. The tolerance observed in the patients who underwent combined transplantation might be associated with a protective phenotype and gene expression of Tregs. However, the mechanism underlying the potential benefits of grafting both organs still needs to be clarified.

There is some indication that the interaction between the higher number of total natural killer cells and Tregs, especially Helios^+^IFN-*γ*^−^ Tregs, probably alleviated graft damage in patients with long-term stable kidney transplantation [60]. With regard to the underlying mechanism, it is possible that the release of TGF-*β* and the cytotoxic T lymphocyte-associated protein 4-mediated interaction of dendritic cells in the lymph nodes with Helios^+^IFN-*γ*^−^ Tregs coexpressing the activation marker HLA-DR promoted long-term allograft tolerance by inhibiting graft-reactive effector cells [60]. In addition, as reported in a previous study, the use of the immunosuppressant sirolimus in combination with RGI-2001 (an activator of natural killer cells) resulted in an increase in the proliferation of Foxp3^+^Helios^+^ Tregs and also reduced the incidence of graft-versus-host disease in patients who underwent allogeneic hematopoietic cell transplantation [61]. Thus, the findings of the included studies indicate that Foxp3^+^Helios^+^ Tregs may be helpful to induce and maintain long-term allograft tolerance.

### 4.3. Infectious Diseases

In elderly pneumonia patients, CD4^+^ T cells from peripheral blood mononuclear cells exhibit reduced Foxp3 and Helios expression in comparison with age- and sex-matched healthy controls [62]. Under *in vitro* conditions, these cells display impaired capacity to upregulate Foxp3 and Helios transcription and lower TGF-*β* and IL-10 production. Further, CD4^+^CD25^+^ T cells from pneumonia patients have been found to secrete significantly lower levels of cytolytic molecules than those from healthy individuals and to also have poor capacity to suppress effector T cell proliferation. Another interesting finding was that the pneumonia severity index in elderly patients was inversely correlated with the proportion of Foxp3^+^ and Helios^+^ cells among CD4^+^ T cells and the level of TGF-*β* [62]. In a mouse model of invasive pneumococcal infection, the level of the immunomodulatory cytokine TGF-*β* in the pneumococcal pneumonia-resistant BALB/c mouse strains was higher than that in susceptible CBA/Ca mouse strains [63]. Additionally, the elevated protein level of TGF-*β* was associated with a rapid rise in Foxp3^+^Helios^+^ Tregs in the lungs of the BALB/c mice. Blocking the induction of Foxp3^+^Helios^+^ Tregs with the short synthetic peptide P17, an inhibitor of TGF-*β*, impaired resistance to pneumococcal infection and contributed to bacterial dissemination. By contrast, adoptive transfer of *in vitro*-induced Foxp3^+^ Tregs in susceptible CBA/Ca mice caused a significant increase in survival and reduced bacterial translocation into the bloodstream [63]. These findings indicate that during invasive pneumococcal infection, Foxp3^+^Helios^+^ Tregs might play a protective role by constraining infection-related inflammation and facilitating resolution of lung tissue damage.

Perinatally HIV-infected children exhibit a significant increase in the frequency of Tregs coexpressing Foxp3 with Helios, rather than with CD25, and selective expansion of CD45RO^+^ (memory) Foxp3^+^Helios^+^ Tregs, regardless of whether antiretroviral therapy is administered [64]. Conversely, the frequency of CD45RO^−^ (naive) Foxp3^+^Helios^+^ Tregs was not significantly different between HIV^+^ and HIV^−^ children. In addition, the increase in the population of memory Foxp3^+^Helios^+^ Tregs in HIV^+^ children was associated with disease progression; this is based on the fact of a decline in the percentage of CD4 cells and CD4 : CD8 ratios and an increase in plasma viremia. Thus, in the case of HIV infection, Foxp3^+^Helios^+^ Tregs may also promote the progression of illness.

Another study assessing the induction and function of inhibitory receptors in Tregs from *Plasmodium vivax*-infected patients demonstrated that circulating Tregs displayed impaired suppressive capacity and proinflammatory features via increased PD-1 expression [65]. Further, *P. vivax* infection resulted in higher expression of CTLA-4 and PD-1 in Tregs and an increase in the frequency of CD4^+^Foxp3^+^ Tregs. Coexpression of CTLA-4 and PD-1 on Tregs was positively correlated with serum bilirubin levels, and PD-1^+^ Tregs had lower levels of Foxp3 and Helios, but higher frequency of T-bet^+^ and IFN-*γ*^+^ cells, than PD-1^−^ Tregs. Thus, in the case of *P. vivax* infection, Foxp3^+^Helios^+^ Tregs might be involved in the damage by pathogens.

### 4.4. Cancer

Alterations in the type of immune cells within tumor tissues can affect the prognosis of cancer patients. At different development stages of primary ovarian cancer, CD4^+^ T cell subsets, but not other infiltrating cells, exhibit dynamic changes, as indicated by an initial Th17 cell response, a slight increase in Th1 cell recruitment in stage II, and then predominant regulatory T cell infiltration at the advanced stage [66]. The tumor-infiltrating Tregs mainly display a Helios^+^ activated and stable phenotype accompanied by demethylation of TSDR. The percentage of intratumoral Foxp3^+^Helios^+^ Tregs was markedly higher in tumor tissue than in peripheral blood, but there was no significant alteration in the different stages of cancer. The recruitment of tumor-infiltrating Tregs, which is probably associated with CCR4/CCL22 interaction, contributed to a specific immunosuppressive microenvironment in the advanced stages of ovarian cancer. Further, T cells in the breast cancer microenvironment exhibited a terminally exhaustive phenotype [67]. Preferential accumulation of the Foxp3^+^Helios^+^ Treg subset was associated with highly immunosuppressive characteristics and coexpression of CTLA-4 and PD-1. Thus, Tregs and their diverse associated immunosuppressive factors create an immune-subversive environment for breast tumor cells and might contribute to an unfavorable prognosis.

The antitumorigenic or protumorigenic effect of Tregs on colorectal cancer (CRC) might be dependent on the different phenotypic characteristics of tumor-infiltrating immune cells. CD4^+^ T cells, which comprise the majority of infiltrating CD3^+^ T cells in CRC tissue, display high coexpression of PD-1/CTLA-4 and PD-1/CD39 [68]. The larger CD4^+^Foxp3^+^Helios^+^ Treg subsets also coexpress high levels of these suppressive molecules. The dual blockade of PD-1/CTLA-4 and PD-1/CD39 by specific neutralizing antibodies might abrogate the exhaustive state of T cells, restore the activation and proliferation of effector T cells, and attenuate the functions of these infiltrating Treg subsets. Additionally, compared to the nontumoral mucosa and peripheral blood of CRC patients, the tumor tissue was characterized by significant accumulation of Helios^high^ Tregs, high levels of TSDR demethylation at the FOXP3 locus, and high levels of OX40 and CD39 expression [69]. These Tregs with multiple activities not only suppressed antitumor immunity mediated by effector T cells but also released IL-17 derived from Th17-like Treg polarization or switched into T follicular regulatory cells, which might impair Tfh response and, thereby, contribute to the establishment and progression of CRC. However, the Helios^+^ subpopulation among CD4^+^Foxp3^+^ T cells in peripheral blood did not significantly differ between non-small-cell lung cancer (NSCLC) patients and healthy donors [70]. Further, the Foxp3^+^Helios^−^ Treg cell population was expanded and mainly mediated immunosuppression in NSCLC patients, and low expression of Helios in infiltrating Tregs was associated with poor survival. Thus, Helios expression in Tregs may differ across tumors.

Although radiotherapy directly damages tumor cells and enhances antitumor immunity, it involves the induction of suppressive immune components, including Tregs, in the tumor sites. According to the small animal radiation research platform, tumor-infiltrating lymphocytes- (TIL-) Tregs from irradiated implanted tumors (B16/F10, RENCA, and MC38) not only exhibited robust proliferation but also upregulated CTLA-4, 4-1BB, and Helios expression and retained their suppressive function [71]. In addition, these expanded Tregs were not dependent of TGF-*β* and IL-33 signaling for their function. Thus, radiotherapy-mediated changes in Tregs and other effector T cells might provide the basis for a selective treatment regimen involving a combination of radiotherapy and Treg depletion therapy, which might optimize the individual antitumor efficacy of each treatment.

Transcriptome analysis based on RNA sequencing showed that splenic Tregs isolated from B16/F10 tumor-bearing Helios WT mice and Helios KO mice exhibited significantly different gene expression than intratumoral Tregs isolated from the corresponding Helios-sufficient and Helios-deficient mice [72]. Genetic reprogramming of Helios-deficient Tregs within the inflammatory tumor site indicated their phenotypic conversion to effector Th cells, as indicated by the upregulation of genes associated with Th cell differentiation and effector T cell activation. Helios-deficient intratumoral Tregs displayed higher levels of GITR/PD-1 and an affinity for tumor-associated self-antigens, but this was not observed in intrasplenic or peripheral lymphoid Tregs. These genetic and phenotypic changes in Helios-deficient Tregs within the tumor microenvironment may provide the basis for understanding the role of Helios as a potential target for improving antitumor immune responses.

## 5. Conclusion

The studies discussed here highlight the presence of a specific phenotype of Foxp3^+^ Tregs coexpressing the Helios transcription factor, which is associated with multiple disorders. The purpose of this review is to help the current understanding of the dynamics of Helios expression, function, and mechanism in Tregs and the role of Foxp3^+^Helios^+^ Tregs in the immune microenvironment in various human diseases. Even though Helios expression has been found to be regulated by a variety of cytokines, surface molecules, and signaling pathways, the underlying molecular mechanism at both the transcription and posttranscriptional levels remains to be further clarified. Overall, molecular markers and phenotypic characteristics of Helios^+^Foxp3^+^ Tregs could be a valuable target in human diseases, and their potential should be explored further in the clinical setting (Table 1). Additionally, an improved understanding of the interactions between Helios^+^Fop3^+^ Tregs and other immune cells, as well as tissue or tumor cells, especially in the tumor microenvironment, would aid in the establishment of improved immunotherapy methods targeting Helios-expressing Tregs.

## Figures and Tables

**Table 1:** Summary of studies that examined the status of Helios in human diseases.

Disease	Change in Foxp3^+^Helios^+^ Treg frequency vs. control	Sample source	Change in Foxp3^+^Helios^+^ Treg functionality	Reference no.
SLE	Increase	PB	Lack effector cytokine production	[52]
RA	Increase	Synovial fluid	Lack IFN-*γ*, TNF, and IL-10 production	[54]
LT	Increase	PB	Not measured	[57]
CLK	Increase	PB	Not measured	[59]
KT	Increase	PB	Stable Foxp3 expression	[60]
HSCT	Increase	PB	Not measured	[61]
Elderly CAP	Decrease	PB	Poorly suppress effector T cell proliferation	[62]
Child HIV	Increase	PB	Not measured	[64]
*P. vivax*	Decrease	PB	Higher expression of CTLA-4 and PD-1	[65]
POC	Increase	Tumor tissue	Not measured	[66]
BC	Increase	Tumor tissue	High levels of CTLA-4 and PD-1 expression	[67]
CRC	Increase	Tumor tissue	High levels of OX40 and CD39 expression	[69]
NSCLC	No change	PB	Not measured	[70]

Treg = regulatory T cell; SLE = systemic lupus erythematosus; RA = rheumatoid arthritis; PB = peripheral blood; LT = liver transplantation; CLK = liver-kidney transplantation; KT = kidney transplantation; HSCT = allogeneic hematopoietic cell transplantation; elderly CAP = elderly community-acquired pneumonia; *P. vivax* = *Plasmodium vivax*; POC = primary ovarian cancer; BC = breast cancer; CRC = colorectal cancer; NSCLC = non-small-cell lung cancer.

## Abbreviations

Tregs: Regulatory T cells
Th: T-helper cell
Tfh: T follicular helper
FOXP3: Forkhead box P3
Breg: Regulatory B cell
ROR*γ*t: Retinoic acid-related orphan receptor-*γ*t
ILCregs: Regulatory innate lymphoid cell
Helios/IKZF2: IKAROS family zinc finger 2
TSDR: Treg-specific demethylated region
TGFBR II: Transforming growth factor beta receptor II
STAT5: Signal transducer and activator of transcription 5
VEGFA/VEGFR2: Vascular endothelial growth factor A/receptor 2
Bcl-2: B cell lymphoma-2
RA: Rheumatoid arthritis
TCZ: Tocilizumab
TNFR-2: TNF receptor-associated factor receptor 2
GITR: Glucocorticoid-induced TNFR family-related protein
SLE: Systemic lupus erythematosus
SSc: Systemic sclerosis
miRNA: MicroRNAs
CTLA-4: Cytotoxic T lymphocyte-associated antigen
PD-1: Programmed death 1
OX40: Also known as Tnfrsf4 (tumor necrosis factor receptor superfamily, member 4)
CD39: Also known as ENTPD1 (ectonucleoside triphosphate diphosphohydrolase-1)
CCR4: C-C motif chemokine receptor 4
CCL22: C-C motif chemokine ligand 22
CRC: Colorectal cancer
NSCLC: Non-small-cell lung cancer
TIL: Tumor-infiltrating lymphocytes.
